# The beneficial use of nitric oxide during cardiopulmonary bypass on postoperative outcomes in children and adult patients: a systematic review and meta-analysis of 2897 patients

**DOI:** 10.1007/s00228-023-03554-9

**Published:** 2023-08-31

**Authors:** Mohamed Abouzid, Yara Roshdy, John Magdy Daniel, Fayed Mohamed Rzk, Ali Ahmed Ali Ismeal, Mohamed Hendawy, Mohammad Tanashat, Marwa Elnagar, Nada Daoud, Alaa Ramadan

**Affiliations:** 1 Department of Physical Pharmacy and Pharmacokinetics, Faculty of Pharmacy, Poznan University of Medical Sciences, Rokietnicka 3 St., 60-806 Poznan, Poland; 2 Doctoral School, Poznan University of Medical Sciences, 60-812 Poznan, Poland; 3 Faculty of Medicine, South Valley University, Qena, Egypt; 4 Faculty of Medicine, Menofia University, Menofia, Egypt; 5 Faculty of Pharmacy, Minia University, Minya, Egypt; 6 Faculty of Pharmacy, Alexandria University, Alexandria, Egypt; 7 Faculty of Medicine, Yarmouk University, Irbid, Jordan; 8 Faculty of Pharmacy, MTI University, Cairo, Egypt; 9 Faculty of Medicine, University of Tripoli, Tripoli, Libya

**Keywords:** Nitric oxide, Cardiopulmonary bypass, CPB

## Abstract

**Purpose:**

Investigate inhaled nitric oxide’s influence on mortality rates, mechanical ventilation and cardiopulmonary bypass duration, and length of stay in the intensive care unit and hospital when administered during cardiopulmonary bypass.

**Methods:**

Following the PRISMA guidelines, we searched four electronic databases (PubMed, EMBASE, Cochrane Library, and Web of Science) up to 4th March 2023. The protocol was registered in the PROSPERO database with ID: CRD42023423007. Using Review Manager software, we reported outcomes as risk ratios (RRs) or mean difference (MD) and confidence intervals (CIs).

**Results:**

The meta-analysis included a total of 17 studies with 2897 patients. Overall, there were no significant differences in using nitric oxide over control concerning mortality (RR = 1.03, 95% CI 0.73 to 1.45; *P* = 0.88) or cardiopulmonary bypass duration (MD = −0.14, 95% CI − 0.96 to 0.69; *P* = 0.74). The intensive care unit days were significantly lower in the nitric oxide group than control (MD = −0.80, 95% CI − 1.31 to −0.29; *P* = 0.002). Difference results were obtained in terms of the length of stay in the hospital according to sensitivity analysis (without sensitivity [MD = −0.41, 95% CI − 0.79 to −0.02; *P* = 0.04] vs. with sensitivity [MD = −0.31, 95% CI − 0.69 to 0.07; *P* = 0.11]. Subgroup analysis shows that, in children, nitric oxide was favored over control in significantly reducing the duration of mechanical ventilation (MD = −4.58, 95% CI − 5.63 to −3.53; *P* < 0.001).

**Conclusion:**

Using inhaled nitric oxide during cardiopulmonary bypass reduces the length of stay in the intensive care unit, and for children, it reduces the duration of mechanical ventilation.

**Supplementary Information:**

The online version contains supplementary material available at 10.1007/s00228-023-03554-9.

## Introduction

Cardiopulmonary bypass (CPB) is a procedure that keeps the body’s blood and oxygen supply flowing during surgery by temporarily replacing the heart and lungs with a machine. CPB may be required for patients with coronary artery bypass grafting, aneurysm surgery, heart transplant, heart valve surgery, or lung transplant. Platelets are activated and consumed as blood travels across the synthetic surfaces of the CPB circuit [1]. In addition to necessitating blood product replacement, platelet activation is a factor in the severe inflammatory response observed with CPB procedures [2]. Postoperative problems such as respiratory failure, renal dysfunction, bleeding issues, cognitive dysfunction, altered liver function, cardiac injury, and multiple organ failure may arise due to this inflammatory cascade [3]. Vascular nitric oxide (NO) bioavailability is decreased by CPB, in part due to NO scavenging (through a deoxygenation reaction in the presence of intravascular hemolysis) [4, 5]. NO regulates the endothelial function and microvascular inflammation and is an endogenous anti-inflammatory mediator [6, 7]. When NO binds to platelet intracellular receptors, it has many functions that inhibit platelet activation and aggregation [8]. In primary and secondary pulmonary hypertension from congenital heart disease and patients having cardiac surgery, inhaled NO has been demonstrated to promote pulmonary vasodilation [9–11]. Currently, NO is being investigated for its potential protective effects against myocardial damage in patients receiving CPB in several randomized trials [12–14]. It is crucial from a therapeutic standpoint to establish the ideal NO dosage range for adult cardiac surgery since many NO-required patients are critically unwell and have failed conventional therapy [15]. In patients susceptible to its effects, prompt therapy with a suitable amount of inhaled NO may eliminate the need for CBP and, in certain circumstances, be life-saving [15]. Currently, several clinical trials on NO have been reported, and results were inconsistent. Furthermore, CPB is utilized for the surgical correction of congenital heart diseases in children [14, 16–18]. Low cardiac output syndrome might occur in this operation and cause multiorgan failure [19]. In adults, it finds application in other conditions, such as heart valve replacement or repair surgeries [3, 20–25]. However, it is worth mentioning that the reported incidence of low cardiac output syndrome varies from 2 to 27% in the adult population. In the pediatric population, reported incidences are between 17 and 67% [26]. Hence, it would be valuable to underscore the distinctions between its utilization in adults and children.

Therefore, we conducted a new systematic review and meta-analysis to compare mortality, length of mechanical ventilation, length of stay in the hospital and intensive care unit, and CBP duration between adults and children receiving inhaled NO during CPB and standard care.

## Methods

We followed PRISMA statement guidelines when reporting this systemic review and meta-analysis [27]. All steps were done in accordance with the Cochrane Handbook of Systematic Review and Meta-analysis of Interventions (version 5.1.0) [28]. This review methods were established prior to the conduct of the review and no significant deviations from the protocol was observed, and it is registered in the PROSPERO database with ID: CRD42023423007. The thorough PRISMA checklist is shown in supplementary file 1.

### Eligibility criteria

We included studies in our review if they satisfied the following criteria:

1. *Population*: patients undergoing cardiopulmonary bypass.
2. *Intervention*: nitric oxide administration during cardiopulmonary bypass.
3. *Comparator*: standard care without nitric oxide administration.
4. *Outcome*:
  (i) Primary outcomes: mortality and length of mechanical ventilation
  (ii) Secondary outcomes: the length of stay in the hospital and intensive care unit (ICU) and CBP duration
5. *Study design*: we included clinical trials and randomized clinical trials.

And we excluded non-published data, reviews, case reports, editorial letters, conference abstracts, study protocols, animal and phantom studies, and patients who had received NO after CBP. No restriction on the language of the study was determined.

### Search strategy

We searched the following electronic medical databases: Embase, PubMed, Web of Science, and Cochrane Library from inception till 4th March 2023. The search query is available in (Supplementary File 2).

### Screening and data extraction

Citations with abstracts were retrieved from the databases and inserted into the Rayyan database. We resolved the duplications prior to screening. Due to many exported studies, every two authors formed a team, and studies were split equally to assess their relevance. In case of a conflict, a third opinion was made to resolve it, and it was based on reading the original article. Next, the full articles were retrieved, and the entire screen of text started to determine the final eligibility of the study for our meta-analysis. Then, we extracted data in duplicate from studies accordingly in a uniform sheet for primary and secondary outcomes.

### Assessment of risk of bias

Two independent authors (Y.R., F.R.) performed the quality assessment of the screened studies using the Risk of Bias 2 (RoB 2) tool [29], and discrepancies were resolved by consensus. The following domains were evaluated individually and graded as “low risk,” “high risk,” or “no information”: randomization processes, deviations from intended intervention, missing outcome data, measuring the outcomes, and selection of reported results.

### Data analysis and synthesis

Statistical analyses were conducted using Review Manager v.5.3. We calculated the pooled risk ratios (RR) and 95% confidence intervals (CI) for the binary outcome of mortality using the random-effects model. It accommodates a larger standard error in the pooled estimate, making it suitable for inconsistent or controversial estimates. We estimated the mean difference (MD) and 95% CI for continuous outcomes of the length of mechanical ventilation, ICU and hospital stay, and CBP duration. If continuous variables were expressed as a median and interquartile range, the mean and standard deviation were computed based on the median, interquartile range, and sample size, as described by Hozo et al. [30]. Regarding the heterogeneity, the chi-square test evaluated statistical heterogeneity among studies. Then, the chi-square statistic was used to calculate *I*-squared. Chi-square with a *P* value less than 0.1 was considered significant heterogeneity. Also, the *I*-square value of more than or equal to 50% indicated high heterogeneity [31]. We used pairwise deletion for missing variables. We performed a subgroup analysis to investigate whether the effect of inhaled NO varied between children and adults. We performed sensitivity analyses to determine the robustness of the effect size by removing one study per time to check the strength of the evidence and ensure the overall results were not altered.

## Results

### Search results and study selection

**Fig. 1:**
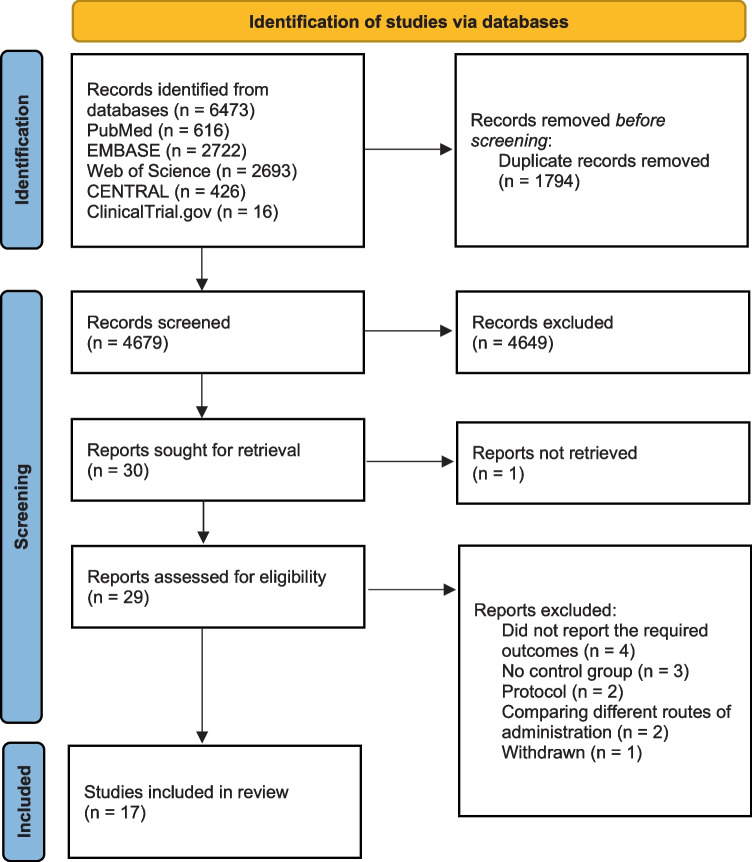
PRISMA chart of the reported studies showing the search selection strategy and exclusion criteria [27]

Through the electronic search, 6473 citations were identified. After excluding 1794 duplicates, 4083 studies were chosen for further evaluation. Through reading the titles and abstracts, 4649 ineligible studies were excluded, and 30 studies were identified as potentially eligible for inclusion and were evaluated by reading the full text. We did not retrieve the full text of a Chinese study due to the language barrier; hence, 29 studies went for full-text reading. Twelve studies were excluded due to the absence of a control group, did not report the required outcomes, compared different routes of NO administration, a withdrawn clinical study, or clinical trial protocol. Finally, 17 studies were eligible for systematic review (*n* = 2897 patients) [3, 13, 14, 16–18, 20–25, 32–36]. Figure 1 shows the process of literature selection and reasons for study exclusion.

### Characteristics of individual studies

The characteristics of the included studies and outcomes are summarized in Tables 1 and 2. These studies vary in the condition of the patients and NO doses. We also split the studies according to the age of the patients. Adults were involved in 10 studies [3, 20–25, 33–35], while seven studies included children, infants, and neonates [13, 14, 16–18, 32, 36].

**Table 1:** Summary of the characteristics of the included studies

**Ref.**	**Study**	**Condition**	**Comparison**	**NO dose (PPM)**	**Main outcomes**	**Source of funding**	**Country**	**Design**	**N**		Age (year)*		**Male**(***n***)		**Female** (***n***)	
									NO	Control	NO	Control	NO	Control	NO	Control
**Adults**																
[21]	Mellgren et al. 1998	Coronary bypass grafting or valve replacement	N	40	NO might decrease the platelet consumption encountered during CPB without having any adverse effect on platelet function	Grants from AGA AB Medical Research Fund and the Swedish Heart Lung Foundation	Sweden	RCT	10	10	73 (5)	69 (3)	8	4	2	6
[22]	Prendergast et al. 1998	Coronary artery bypass graft surgery	N	20	Comparison of arterial oxygenation, cardiac output, and mean pulmonary arterial and systemic arterial pressures	A grant from Chest Heart and Stroke, Scotland	UK	RCT	20	20	67.3 (6.7)	65.4 (6.6)	16	17	4	3
[3]	Gianetti et al. 2004	Aortic valve replacement combined with aortocoronary bypass	No additional inhalation treatment	20	• Administration of NO via inhalation before, during, and after surgery may positively affect the pattern of release of markers of myocardial injury and left ventricular dysfunction, which significantly reduces clinical morbidity • NO by-products in peripheral plasma samples show a significant increase during NO therapy, beginning with the ventilatory phase and continuing throughout the extracorporeal circulation	Not mentioned	Italy	Prospective, randomized, nonblinded study	14	15	70 (13)	69 (10)	9	8	5	7
[33]	Taylor et al. 2004	Moderate to severe acute lung injury	N	5	Days patients were alive and not receiving assisted breathing to day 28 was not different in the placebo and intervention	Ohmeda PPD/INO Therapeutics Inc	United States	Multicenter, randomized, placebo-controlled study, with blinding of patients, caregivers, data collectors, assessors of outcomes, and data analysts (triple blind)	192	193	50 (12)	50 (12)	100	104	92	89
[34]	Chung et al. 2005	Coronary artery bypass grafting	Routine CPB	20	Effects of NO and iloprost on CPB-induced changes in platelet and leukocyte numbers which shows a reduction in postoperative bleeding with the use of (iloprost and NO)	Canadian Institutes of Health Research and Research Support program of the University of Munich and the Wamsler Foundation, section New York	Canada	Pilot clinical study	8	18	64 (8)	61 (12)				
[23]	Fattouch et al. 2006	Severe mitral stenosis and pulmonary hypertension	Intravenous vasodilator	400 diluted	• NO and iPGI2 were effective in treating pulmonary hypertension • iPGI2 has more benefits than NO	Not mentioned	Italy	Prospective randomized double-blind clinical trial	21	18	65 (9)	64 (7)				
[24]	Li et al. 2018	Multiple valve replacement surgery, mostly due to rheumatic	N	80	Using NO decrease the incidence of postoperative acute kidney injury and reduce the transition to stage 3 of CKD	National Natural Science Foundation of China, the Xijing Hospital Foundation, the National Key Technology Research and Development Program of the Ministry of Science and Technology of China	China	Prospective RCT	117	127	48.7 (9.5)	48.4 (8.6)	52	52	65	75
[35]	Kamenshchikov et al. 2020	Moderate risk of renal complications who underwent elective cardiac surgery with cardiopulmonary bypass	Without NO	40	NO administration was associated with decreased acute kidney injury incidence	None	Russia	Prospective RCT	48	48	64 (2.7)	63 (2.8)	30	31	18	17
[25]	Pichugin et al. 2020	Heart valves surgery with cardiopulmonary bypass (CPB) with high pulmonary hypertension	Without NO	20	• Developed technology for inhaled NO in surgery with CPB provides a clinically marked protective effect on the heart and lungs • The effectiveness of NO’s protective action depends on its administration duration and is most pronounced when used during the entire operation, including CPB time	None	Russia	Random prospective study	30	30	58.6 (1.4)	54.1 (1.4)	13	12	17	18
[20]	Nakane et al. 2021	Pulmonary hypertension underwent valvular surgery	Without NO	20	Inhaled NO therapy ameliorated pulmonary hypertension and improved postoperative respiratory, coagulation, and renal functions in adult valve surgeries	None	Japan	RCT	30	65	72.8 (9.5)	73.6 (8.1)	19	40	11	25
**Children**																
[17]	Miller et al. 2000	Corrective surgery for congenital heart disease	N	10	Observed reduction of pulmonary hypertensive crises	National Health and Medical Research Council	USA	RCT	63	61	0.25 (0.083)	0.167 (0.063)	36	28	27	33
[14]	Checchia et al. 2013	Complete repair of tetralogy of Fallot	Placebo	20	The children who received iNO during CPB had an improved postoperative course, as demonstrated by a shorter length of stay in the pediatric CICU, a shorter duration of mechanical ventilation requirement, and improved indexes of myocardial injury and function	Research grant from Ikaria, Inc. and National Institutes of Health/National Institute of General Medical Sciences grant K08GM084143-01	USA	Prospective, randomized, blinded, placebo-controlled study	8	8	0.523 (0.307)	0.592 (0.312)	7	4	1	4
[13]	Christopher et al. 2016	Cardiac surgery with CPB	Standard Care	20	Administration of NO reduce the incidence of LCOS	None	Australia and New Zealand	Prospective RCT	101	97	1.15 (1)	0.49 (0.58)	61	55	40	42
[18]	Elzein et al. 2020	Hypoplastic left heart syndrome or variant who underwent Norwood procedure in the first few days of life	Without NO	40	Systemic administration of NO during the Norwood procedure has myocardial protective effects (lower troponin levels), but we observed no effect on postoperative recovery	Mallinckrodt Pharmaceuticals	USA	Prospective randomized blinded controlled trial	12	12	0.016 (0.005)	0.016 (0.005)	8	7	4	5
[36]	Niebler et al. 2020	CPB surgery	N	20	• NO added to the sweep gas of the oxygenator during CPB in infants did not have an appreciable effect on the preservation of platelet count, platelet responsiveness to agonist, or clinical outcomes • Methemoglobin levels were increased after receiving NO but were far below a toxic level of 15%	Grant from the Clinical and Translational Science Institute of Southeast Wisconsin. The investigational product (NO) and delivery device (iNOmax) were provided by the manufacturer (Mallinckrodt Pharmaceuticals; Bedminster, NJ, USA)	USA	Pilot randomized controlled trial	18	22	0.276 (0.213)	0.308 (0.253)	9	13	9	9
[16]	Kolcz et al. 2022	Single ventricle congenital heart defect	FONTAN vs. FONTAN NO	20	NO inhaled into the oxygenator during CPB can improve short‑term clinical outcomes. It shortens intubation time and intensive care time. It reduces inflammatory response, improves myocardial and lung protection, and diminishes metabolic stress in patients with a single ventricle undergoing Fontan surgery	None	Poland	Prospective randomized study	48	49	2.5 (0.7)	2.6 (0.6)	22	21	26	28
[32]	Schlapbach et al. 2022	Heart surgery	N	20	The use of NO via CPB did not significantly affect the number of ventilator-free days	Mallinckrodt Pharmaceuticals and EKU Electronics provided NO delivery devices to study centers but did not involve in study design, analyses, or interpretation of the results	Australia and Switzerland	Multicenter, randomized, double-blind, parallel-group trial	679	685	0.261 (0.079)	0.272 (0.091)	413	368	266	317

*Reported as mean (standard deviation)

**Table 2:** Summary of the primary and secondary outcomes of the included studies

**Ref.**	**Study**	**N**		**Death** (***n***)		Mechanical ventilation (hours)*		Hospitals stay (days)*			ICU stay (days)*		Cardiopulmonary bypass duration (min)*	
		NO	Control	NO	Control	NO	Control	NO	Control	NO		Control	NO	Control
**Adults**														
[21]	Mellgren et al. 1998	10	10										73 (5)	80 (8)
[22]	Prendergast et al. 1998	20	20			67 (26.4)	85 (29.1)	9.9 (1.3)	10.6 (1.3)	4.3 (0.9)		5 (1.3)	66.7 (7.3)	68.3 (7.2)
[3]	Gianetti et al. 2004	14	15											
[33]	Taylor et al. 2004	192	193	44	39									
[34]	Chung et al. 2005	8	18							0.9 (0.1)		1 (0.2)		
[23]	Fattouch et al. 2006	21	18	1	1			9 (2.8)	14 (6)	2 (0.5)		3.3 (1.6)	72 (14)	99 (15)
[24]	Li et al. 2018	117	127	3	8	24.4 (0.9)	24.6 (1)	10 (0.5)	10 (0.7)	2.8 (0.3)		2.8 (0.3)		
[35]	Kamenshchikov et al. 2020	48	48	0	1	9 (2)	10 (4)	10 (2)	12 (0.6)	0.9 (0.1)		1.3 (0.2)		
[25]	Pichugin et al. 2020	30	30										104.8 (33.2)	98.6 (37.1)
[20]	Nakane et al. 2021	30	65	0	6	23 (25)	62 (134)	27 (19)	24 (11)	6.3 (5.9)		6.4 (6.4)		
**Children**														
[17]	Miller et al. 2000	63	61	5	3									
[14]	Checchia et al. 2013	8	8			8.4 (7.6)	16.3 (6.5)	5.6 (2.5)	5.1 (1.2)	2.2 (0.8)		3.3 (1.6)	118 (31)	128 (36)
[13]	Christopher et al. 2016	101	97			20 (8.8)	24 (12.8)	9 (1.8)	12 (2.3)	2 (0.6)		3 (0.8)	131.5 (16.42)	107 (4.5)
[18]	Elzein et al. 2020	12	12					21.7 (4.4)	18.6 (3.8)				143 (17.1)	150.7 (20.36)
[36]	Niebler et al. 2020	18	22	0	0	45.7 (27.9)	43 (28.9)	15.1 (8.6)	17.5 (5.1)				125.3 (14.13)	121.6 (16.08)
[16]	Kolcz et al. 2022	48	49			9.3 (1.6)	13.9 (3.7)	15.6 (2.1)	17.2 (4.2)	3.4 (0.5)		4.3 (1)	95.6 (18.3)	98.5 (13.1)
[32]	Schlapbach et al. 2022	679	685					9 (1.9)	9.1 (1.9)	3 (0.7)		3 (0.7)		

*Reported as mean (standard deviation)

### Risk of bias and quality assessment

**Fig. 2:**
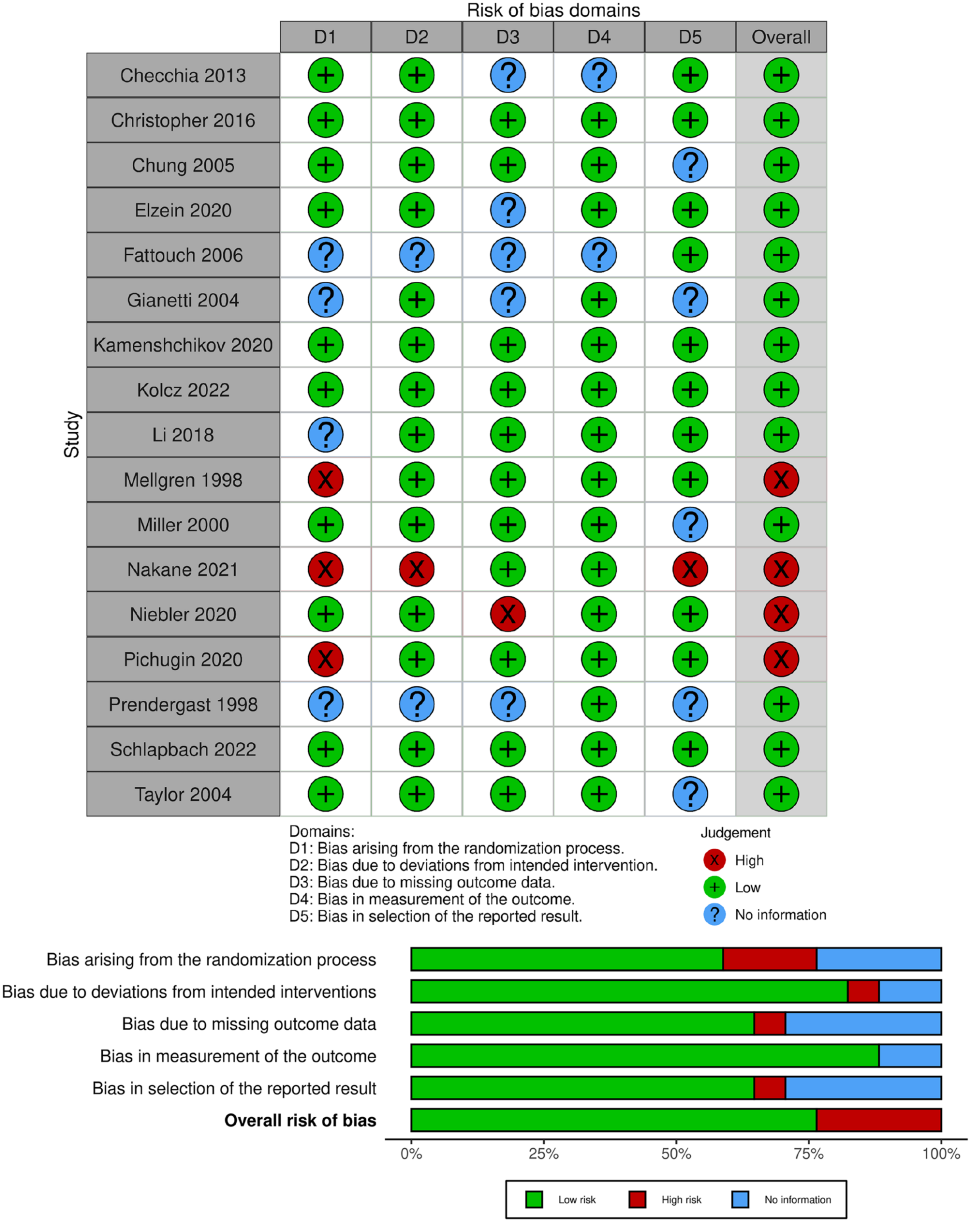
Risk of bias assessment is represented in traffic light plot and summary plot according to the Cochrane risk-of-bias tool, created using robvis [41]

Most of our included studies (13 out of 17) showed a low risk of overall bias, and only four articles showed a high risk of bias. The quality evaluation is shown in Fig. 2.

### Outcomes

#### Mortality

**Fig. 3:**
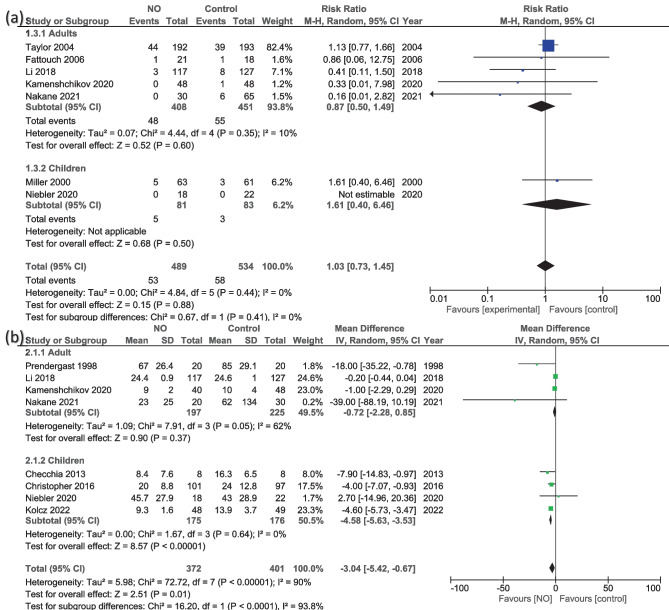
Random-effects models of the risk ratio and mean differences for **a** mortality and **b** duration of mechanical ventilation (hours)

The overall effect of the analysis of seven studies [17, 20, 23, 24, 33, 35, 36] showed an insignificant risk ratio between the NO group and the experimental (RR = 1.03, 95% CI 0.73 to 1.45; *P* = 0.88), with no heterogeneity (*I*^2^ = 0%, *P* = 0.44). Subgroup analysis also showed no significant differences in adult (RR = 0.87, 95% CI 0.50 to 1.49; *P* = 0.60) or children’s groups (RR = 1.61, 95% CI 0.40 to 6.46; *P* = 0.50) (Fig. 3a).

#### Duration of mechanical ventilation (hours)

In eight studies [13, 14, 16, 20, 22, 24, 35, 36], significant differences were observed with regard to the duration of mechanical ventilation between the two groups (MD = −3.04, 95% CI − 5.42 to −0.67; *P* = 0.01), with high heterogeneity (*I*^2^ = 90%, *P* < 0.001) (Fig. 3b). The heterogeneity was not resolved. Subgroup analysis also showed no significant differences in adults (MD = −0.72, 95% CI − 2.28 to 0.85; *P* = 0.37), with heterogeneity (*I*^2^ = 62%, *P* = 0.05). Even though the heterogeneity reduced to *I*^2^ = 48% and *P* = 0.15 after excluding Prendergast et al. [22], the results did not change, even for the overall results. However, the children group favored NO over the control (MD = −4.58, 95% CI − 5.63 to −3.53; *P* < 0.001), with no heterogeneity (*I*^2^ = 0%, *P* = 0.64) (Fig. 3b).

#### ICU time (days)

**Fig. 4:**
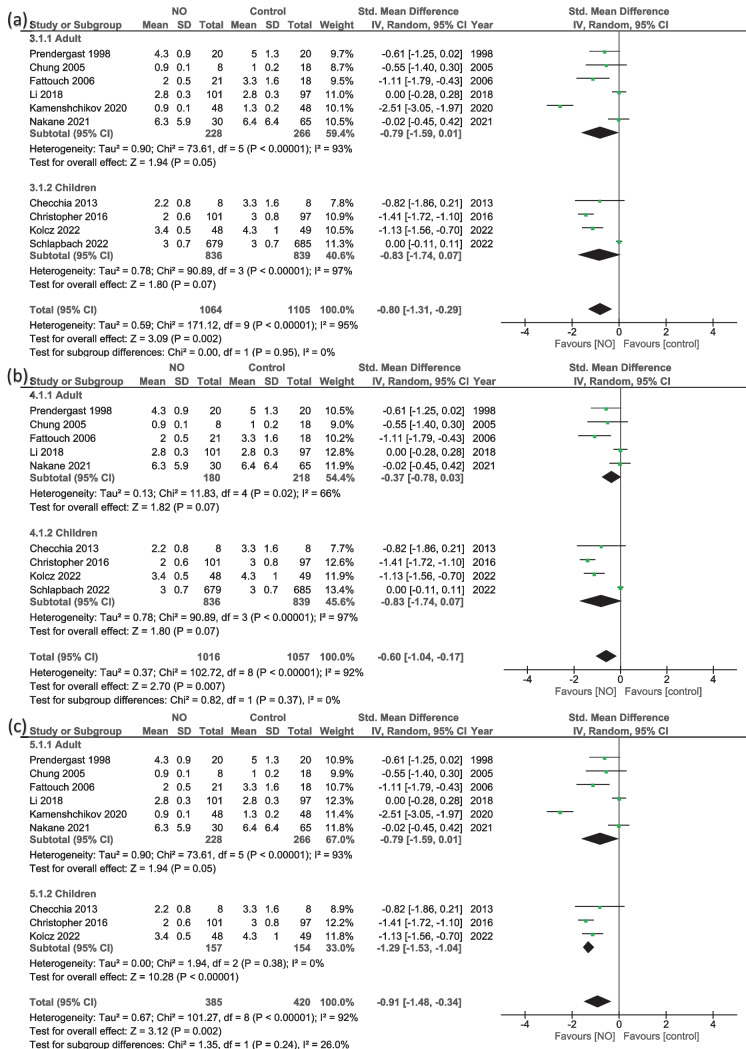
Random-effects models of the mean difference for the length of stay in ICU (days), **a** overall results, **b** sensitivity analysis for adults, and **c** sensitivity analysis for children

The overall effect of ten studies [13, 14, 16, 20, 22–24, 32, 34, 35] showed that the number of days in the ICU was significantly lower in the NO group than control (MD = −0.80, 95% CI − 1.31 to −0.29; *P* = 0.002), with high heterogeneity (*I*^2^ = 95%, *P* < 0.001) (Fig. 4a). In adults, the results were in the trending line of significance to favor NO as well (MD = −0.79, 95% CI − 1.59 to 0.01; *P* = 0.05) with high heterogeneity (*I*^2^ = 93%, *P* < 0.001) (Fig. 4a). However, after excluding Kamenshchikov et al. [35], the results became insignificant (MD = −0.37, 95% CI − 0.78 to 0.03; P = 0.07), and the heterogeneity reduced to *I*^2^ = 66% and *P* = 0.02) (Fig. 4b). In children, the results were insignificant (MD = −0.83, 95% CI − 1.74 to 0.07; *P* = 0.07) with high heterogeneity (*I*^2^ = 97%, *P* < 0.001) (Fig. 4a); however, after excluding Schlapbach et al. [32] (the highest number of patients), the results significantly favored NO (MD = −1.29, 95% CI − 1.53 to −1.04; *P* < 0.001) with no heterogeneity (*I*^2^ = 0%, *P* = 0.38) (Fig. 4c).

#### Hospital stay (days)

**Fig. 5:**
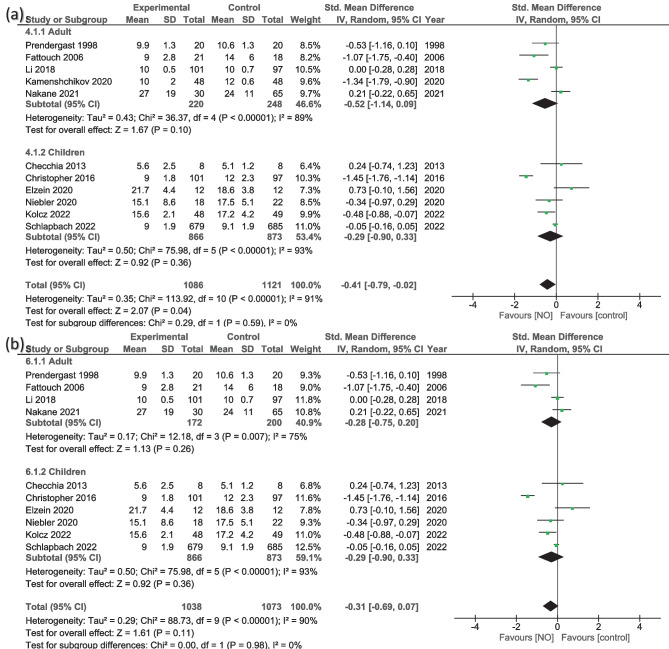
Random-effects models of the mean difference for the length of stay in hospital (days), **a** overall results and **b** sensitivity analysis for adults

Analyzing 11 studies [13, 14, 16, 18, 20, 22–24, 32, 35, 36] showed that hospital stay was significantly less in the NO group (MD = −0.41, 95% CI − 0.79 to −0.02; *P* = 0.04), with high heterogeneity (*I*^2^ = 91%, *P* < 0.001) (Fig. 5a). However, excluding Kamenshchikov et al. [35] from the adult group will show overall insignificant results (MD = −0.31, 95% CI − 0.69 to 0.07; *P* = 0.11) (Fig. 5b); similarly, if we remove any of these studies from the children group (Christopher et al. [13], Kolcz et al. [16], or Schlapbach et al. [32]), it will result in an insignificant reduction in the hospital stay, and the heterogeneity will remain high. Subgroup analysis also showed similar insignificant results in adults (MD = −0.52, 95% CI − 1.14 to 0.09; *P* = 0.10) and children (MD = −0.29, 95% CI − 0.90 to 0.33; *P* = 0.36), with high heterogeneity of *I*^2^ = 89% and *P* < 0.001 and *I*^2^ = 93% and *P* < 0.001, respectively (Fig. 5a).

#### CBP duration (minutes)

**Fig. 6:**
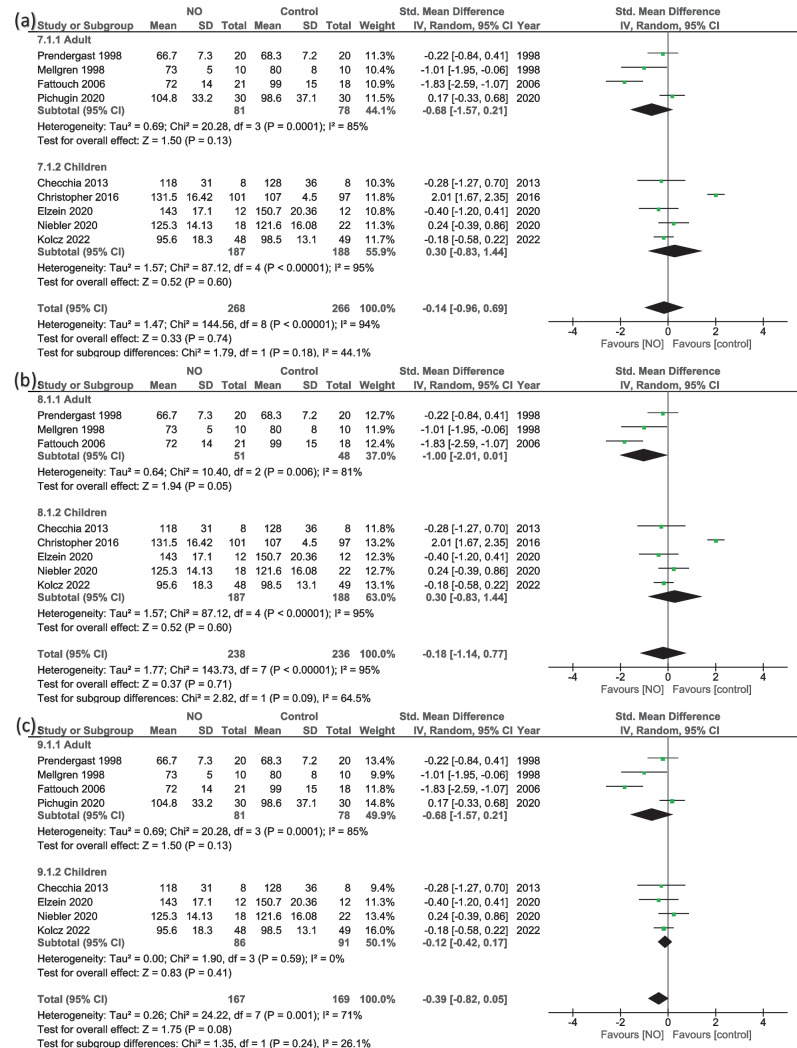
Random-effects models of the mean difference for the duration of cardiopulmonary bypass (minutes), **a** overall results, **b** sensitivity analysis for adults, and **c** sensitivity analysis for children

The overall effect of the analysis of nine studies [13, 14, 16, 18, 21–23, 25, 36] showed that NO did not significantly influence the changes in CBP duration (MD = −0.14, 95% CI − 0.96 to 0.69; *P* = 0.74), with high heterogeneity (*I*^2^ = 94%, *P* < 0.001) (Fig. 6a). Subgroup analysis shows that neither adults nor children significantly reported differences in CBP duration in the two groups (MD = −0.68, 95% CI − 1.57 to 0.21; *P* = 0.13; and MD = 0.30, 95% CI − 0.83 to 1.44; *P* = 0.60, respectively) (Fig. 6a). Heterogeneity was high in the two groups. Removing Pichugin from the adults’ analysis showed a trending line toward significance to favor NO (MD = −1.00, 95% CI − 2.01 to 0.01; *P* = 0.05), with high heterogeneity (*I*^2^ = 81%, *P* = 0.006) (Fig. 6b). Sensitivity in the children group was resolved (*I*^2^ = 0%, *P* = 0.59) by excluding Christopher et al. [13] (largest sample size), and the results remained insignificant (MD = −0.12, 95% CI − 0.42 to 0.17; *P* = 0.41) (Fig. 6c).

### Publication bias for included studies

**Fig. 7:**
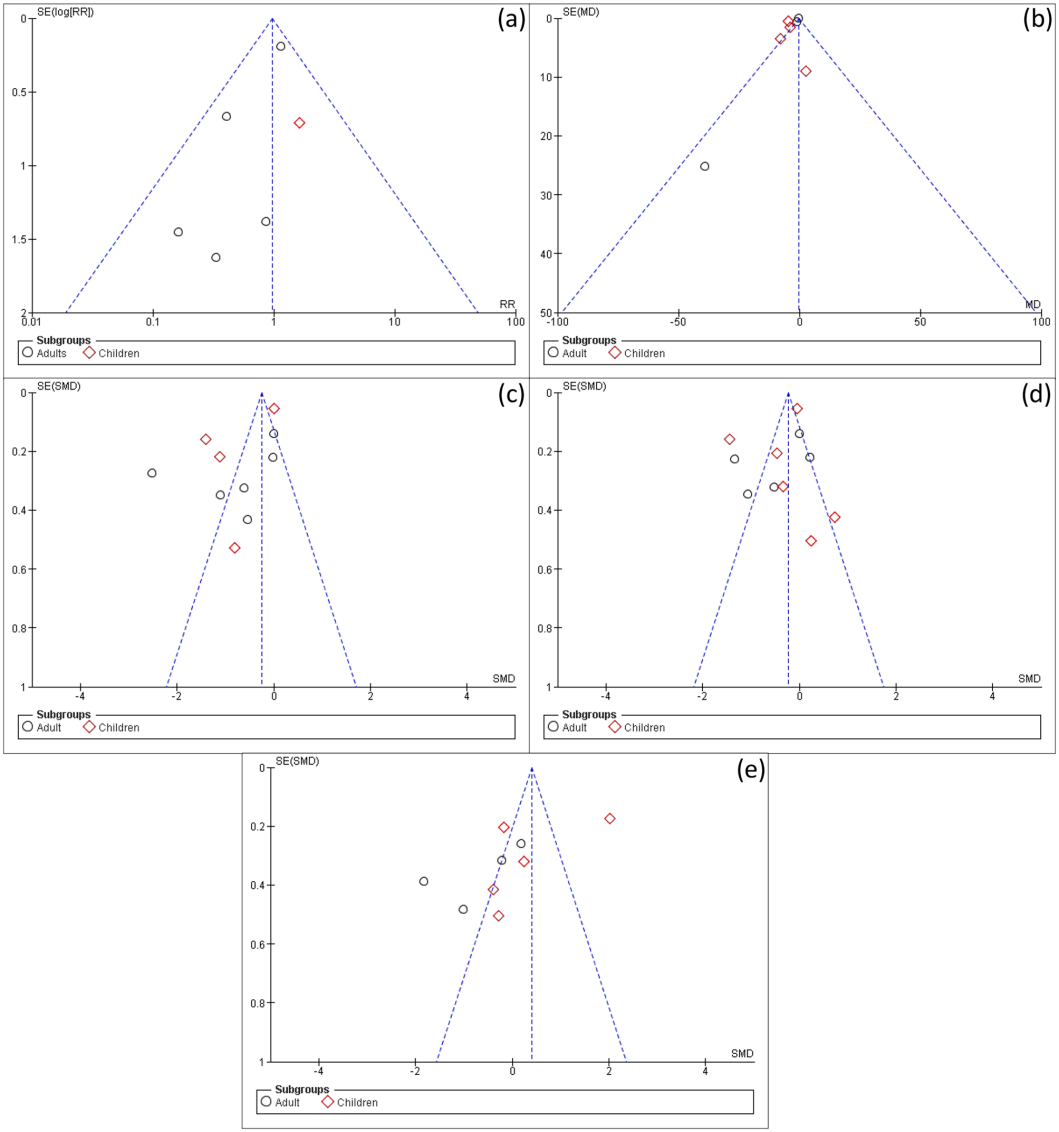
Funnel plot for possible publication bias, **a** mortality, **b** mechanical ventilation, **c** ICU stay, **d** hospital stay, and **e** CBP duration

We found asymmetry in the funnel plots assessing studies of mechanical ventilation, ICU stay, hospital stay, and CBP duration, as shown in Fig. 7, indicating the possibility of publication bias due to the insufficient literature and the heterogeneity.

## Discussion

This systematic review and meta-analysis revealed a significant difference in the outcomes of patients who received inhaled NO during CBP compared to those who did not. The results indicate that administering inhaled NO is associated with overall lower ICU and hospital stay and lower duration of mechanical ventilation, especially in children. NO did not affect mortality rates or CBP duration. Sensitivity analysis showed insignificant results for the influence of NO on reducing ICU time for children and overall hospital stay. Hence, we suggest further studies on a larger sample size for more robust evidence of the beneficial impact of NO.

In children, NO was introduced to CBP based on two trials involving 16 [14] and 198 children [13], respectively. The former showed its beneficial effect on decreasing ICU time and shorter duration of mechanical ventilation, while the latter reported a lower incidence of low cardiac output syndrome in children < 2 years. However, the results reported by previously published large trials with 1364 children > 2 years showed no significant difference in death within 28 days or ventilator-free days between those who received nitric oxide delivered into CPB and those who received care [32]. According to our study, we have strong evidence that the children group favored NO over control concerning the mortality rate and duration of mechanical ventilation. During the sensitivity analysis, excluding large trials [32] altered the results of the length of stay in the ICU, and sensitivity analysis of hospital stay provided insignificant results.

In adults, in a study by Tylor et al. [33], the authors highlighted the lack of long-term benefits despite initial improvements in oxygenation upon using NO, which was observed in this analysis, and NO did not influence the mortality rate, duration of mechanical ventilation or length of ICU stay, hospital stay (through sensitivity analysis), and CBP duration in this group.

Compared to a previous meta-analysis with five studies and 579 patients [37], the authors reported no association between NO therapy and the hospital or ICU stay length. We obtained similar results during sensitivity analysis. It is worth mentioning that the author’s main aim was to evaluate the relative risk of acute kidney injury after NO therapy, which was not necessarily administered during CPB. Our inclusion criteria determined only two studies eligible for renal replacement therapy analysis; however, we did not see significant differences (RR = 0.51, 95% CI 0.13 to 2.02; *P* = 0.34).

Furthermore, a recent meta-analysis by Elnaiem et al. [38] investigated six studies. It included a total of 1666 children who were undergoing cardiac surgery and showed comparative results to our study in terms of reduced time on mechanical ventilation when using NO during CBP. In addition, they showed a reduction in postoperative levels of IL-6 and tumor necrosis factor-alpha (*P* < 0.001 and *P* = 0.05, respectively). The agreement of the results between the studies suggests the beneficial use of NO, particularly for children.

A potential rationale was previously presented by Lincoln et al. [39]. The authors have linked the disparities in acute respiratory distress syndrome between children and adults to the continuing progression of postnatal lung development until reaching adult stature. This process involves an intersection of factors that govern the regulation of postnatal lung maturation, as well as mechanisms related to inflammation, apoptosis, alveolar fluid clearance, and tissue repair. Consequently, a distinct underlying framework of genetic and protein expression exists in children when compared to adults. Building upon this premise, Hunt et al. [40] proposed a hypothesis. Recognizing that the disease state is not uniform across all age groups, the authors proposed that the response to therapies, specifically inhaled NO, might exhibit variations.

## Strength and limitations

One of the main strengths of this study is the large sample size, which increases the reliability of the findings. Additionally, the use of meta-analytic techniques allowed us to pool data from multiple studies and synthesize the results statistically rigorously. This helped increase the study’s power and reduce the risk of type I errors. However, some limitations to this study should be noted. First, some analyses had mild to high heterogeneity, which could limit the generalizability of the findings even when using the random model effect. This heterogeneity could be due to differences in patient populations, clinical conditions, surgical techniques, duration, and postoperative management strategies. Some included studies showed a high risk of bias. Finally, the present study did not report some competing endpoints, such as renal replacement therapy, the impact of different doses—most doses were 20 PPM—or the duration of NO.

## Conclusion

This systematic review and meta-analysis showed that inhaled nitric oxide during cardiopulmonary bypass lowered the overall length of stay in the intensive care unit and might lower the duration of mechanical ventilation in children. Nitric oxide did not influence the mortality rates, hospital stay, or cardiopulmonary bypass duration, suggesting additional high-quality studies to validate these results.

### Supplementary Information

Below is the link to the electronic supplementary material.

Supplementary file1 (DOCX 32 KB)

Supplementary file2 (PDF 94 KB)

## Data Availability

All data generated or analyzed during this study are included in this published article and its supplementary information file.
