# Supplementary material for: The beneficial use of nitric oxide during cardiopulmonary bypass on postoperative outcomes in children and adult patients: a systematic review and meta-analysis of 2897 patients
Source: Eur J Clin Pharmacol. 2023 Aug 31;79(11):1425–42. doi: 10.1007/s00228-023-03554-9 (PMC10618325; doi:10.1007/s00228-023-03554-9)
Supplement: Supplementary file 2 — Supplementary file2 (PDF 94 KB) [file 228_2023_3554_MOESM2_ESM.pdf]

Embase: 2722

('cardiopulmonary bypass'/exp OR 'systemic pulmonary shunt'/exp OR 'cardiopulmonary bypass':ab,ti OR 'heart-lung bypass':ab,ti OR 'heart lung bypass':ab,ti OR 'heart-lung bypasses':ab,ti OR 'cardiopulmonary bypasses':ab,ti OR fontan:ab,ti OR 'mitral valve':ab,ti OR 'cardiac surgery':ab,ti OR 'lung transplantation'/exp OR 'lung transplantation':ab,ti OR 'lung grafting':ab,ti OR 'heart surgery'/exp) AND ('nitric oxide'/exp OR 'nitric oxide':ab,ti) AND ('randomized controlled trial'/exp OR 'controlled clinical trial'/exp OR randomized:ti,ab OR placebo:ti,ab OR 'drug therapy':lnk OR randomly:ti,ab OR trial:ti,ab OR groups:ti,ab)

Pubmed: 616

("Cardiopulmonary Bypass"[Mesh] OR Cardiopulmonary Bypass[tw] OR Heart-Lung Bypass[tiab] OR Heart Lung Bypass[tiab] OR Heart-Lung Bypasses[tiab] OR Cardiopulmonary Bypasses[tiab] OR "Lung Transplantation"[Mesh] OR Lung Transplantation*[tiab] OR Lung Grafting*[tiab] OR Fontan [tiab] OR “mitral valve”[tiab] OR “cardiac surgery”[tiab]) AND ("Nitric Oxide"[Mesh] OR "Nitric Oxide"[tiab]) AND ("randomized controlled trial"[pt] OR "controlled clinical trial"[pt] OR randomized[tiab] OR placebo[tiab] OR "drug therapy"[sh] OR randomly[tiab] OR trial[tiab] OR groups[tiab])

Web of science: 2693

ALL=(cardiopulmonary bypass OR systemic pulmonary shunt OR cardiopulmonary bypass OR heart-lung bypass OR heart lung bypass OR heart-lung bypasses OR cardiopulmonary bypasses OR fontan OR mitral valve OR cardiac surgery OR lung transplantation OR lung transplantation OR lung grafting) AND ALL=(nitric oxide) AND ALL=(randomized controlled trial OR controlled clinical trial OR randomized OR placebo OR drug therapy OR randomly OR trial OR groups)

CENTRAL: 426

#1	(cardiopulmonary bypass)	Limits	7647
#2	heart-lung bypass	Limits	328
#3	heart-lung bypasses	Limits	15
#4	cardiopulmonary bypasses	Limits	119
#5	lung transplantation	Limits	2791
#6	lung transplantations	Limits	54
#7	lung grafting	Limits	445
#8	fontan	Limits	341
#9	mitral valve	Limits	2540
#10	cardiac surgery	Limits	24226
#11	{OR #1-#10}	Limits	31608
#12	nitric oxide	Limits	9093
#13	#11 and #12	Limits	426

ClinicalTrial.gov: 16

Nitric oxide and cardiopulmonary bypass
